# Clonality drives structural patterns and shapes the community assemblage of the Mediterranean *Fagus sylvatica* subalpine belt

**DOI:** 10.3389/fpls.2022.947166

**Published:** 2022-09-16

**Authors:** Luigi Saulino, Angelo Rita, Marina Allegrezza, Maurizio Zotti, Valentina Mogavero, Giulio Tesei, Silvia Montecchiari, Emilia Allevato, Marco Borghetti, Giuliano Bonanomi, Antonio Saracino

**Affiliations:** ^1^Dipartimento di Agraria, Università degli Studi di Napoli Federico II, Naples, Italy; ^2^Dipartimento di Science Agrarie, Alimentari ed Ambientali, Università Politecnica delle Marche, Ancona, Italy; ^3^Scuola di Scienze Agrarie, Forestali, Alimentari e Ambientali, Università della Basilicata, Potenza, Italy

**Keywords:** treeline ecotone, Krummholz, layering, endemic species, microclimatic refugia, environmental heterogeneity, glacial refugia

## Abstract

Past anthropogenic disturbances lowered the altitudinal distribution of the Mediterranean *Fagus sylvatica* forests below 2,000 m a.s.l. Accordingly, our current understanding of the southern distribution range of *F. sylvatica* forests is restricted to managed stands below this elevation, neglecting relic forests growing above. This study has shed light on the structure and species assemblage of an unmanaged relict subalpine *F. sylvatica* stand growing within the core of its southernmost glacial refugia and at its highest species range elevation limit (2,140 m a.s.l.) in southern Apennines (Italy). Here, tree biometric attributes and understory species abundances were assessed in eight permanent plots systematically positioned from 1,650 to 2,130 m a.s.l. In the subalpine belt, *F. sylvatica* had formed a dense clonal stem population that was layered downward on the steepest slopes. The density and spatial aggregation of the stems were increased, while their stature and crown size were decreased. Above 2,000 m, changes in tree growth patterns, from upright single-stemmed to procumbent multi-stemmed, and canopy layer architecture, with crowns packed and closer to the floor, were allowed for the persistence of understory herbaceous species of biogeographic interest. Clonal layering represents an adaptive regeneration strategy for the subalpine belt environmental constraints not previously recognized in managed Mediterranean *F. sylvatica* forests. The clonal structure and unique species assemblage of this relic forest highlight the value of its inclusion in the priority areas networks, representing a long-term management strategy of emblematic glacial and microclimatic refugia.

## Introduction

Climate change and environmental disturbances resulting from anthropogenic activity have had a great impact on high-elevation forest ecosystems and their biota, both regionally and globally (Lenoir and Svenning, 2015; Sánchez-Salguero et al., 2017). An accelerated loss of biodiversity, changes in habitat suitability, and species range shifts are the most significant impacts of climate warming (Randin et al., 2009; Lenoir and Svenning, 2015; Sánchez-Salguero et al., 2017). In this context, Mediterranean mountains are considered “phylogeographical hotspots” refugia (Médail and Diadema, 2009), wherein the cumulative effects of past climate events and the plurimillennial pervasive human pressure have led us to consider these sites as Anthropocene refugia (Monsarrat et al., 2019) rather than merely glacial or climatic refugia (Médail and Diadema, 2009). Throughout the Mediterranean Basin, relicts of old-growth mountain forests preserved by human disturbances are mainly restricted to high elevations in the southern glacial refugia. Here, unique sets of species can still be found (Padullés Cubino et al., 2021b), even at their geographical distribution limit. The understory community species assemblage is shaped by stand structure (Ali, 2019), soil attributes (Jiménez-Alfaro et al., 2018; Weigel et al., 2018), local topoclimatic conditions (Geiger et al., 1995), and increasing isolation with an elevation of mountains, which favors speciation and endemism (Steinbauer et al., 2016).

In Italy, the Apennines Mountains are currently dominated by European beech (*Fagus sylvatica* L.), and its distribution is subject to a repeated range of contractions and re-expansion cycles, following the cycle of European glacial and interglacial periods dated 13–10 cal. kyr BP (Magri et al., 2006). Below a latitude of 43° N, the consistency and altitudinal position of *F. sylvatica* before anthropogenic disturbances may have been climatically controlled by a dry period around 4,200 years ago, the so-called 4.2 ka BP megadrought (Sevink et al., 2019; Di Rita et al., 2022). The current *F. sylvatica* high-elevation limit on the Apennines occurs at lower elevations and higher temperatures than the assessed global climatic treeline distribution (Paulsen and Körner, 2014). Since its range distribution is confined below the climatic treeline, *F. sylvatica*'s upper elevation limit across the Apennines is more properly a species range limit (Körner, 2021), where past anthropogenic disturbances (Bonanomi et al., 2018) are prevailed on the eco-physiological limitations to the growth processes commonly related to the freezing tolerance (Körner, 2012a). Apennine beech forests are also known to be prominent examples of human-shaped forest landscapes, where the forest cover was manipulated starting from the late Holocene (Compostella et al., 2013; Benatti et al., 2019; Morales-Molino et al., 2021). For centuries, along the Italian Apennines' mountain belt (from a latitude of 38° to 44° N), silviculture has been involved mostly in coppicing with the removal of the larger shoots from the stools (Coppini and Hermanin, 2007). Therefore, the current stand structure of beech forests reflects the coppicing and logging legacies, which largely affect the near-ground microclimate pattern and community assembly (Campetella et al., 2011; Scolastri et al., 2017). At the same time, anthropogenic pressures have also markedly reduced the *F. sylvatica* species range elevation limit (Bonanomi et al., 2018, 2020). In the southern Apennine beech stands, the associated understory vegetation assembly may therefore represent a hotspot for narrow-range species, that is, species that migrate only over short distances in the mountains (Willner et al., 2009; Jiménez-Alfaro et al., 2018). This has given rise to glacial refugia that act as a source of beta diversity and promote significant spatial taxonomic turnover (Padullés Cubino et al., 2021a).

In high-elevation environments, clonality, that is, vegetative multiplication resulting from the emergence of a new plant genetically identical to its ancestors but potentially independent with respect to growth and reproduction, is widespread among plants (Klimešová et al., 2021). Clonality is a functional trait that confers important ecological advantages, such as the foraging response of plants and the allocation of resources between ramets, enabling the avoidance of deterministic and stochastic environmental perturbations (Herben et al., 2015). The clonal propagation strategy allows for the persistence of populations in the long term, affecting the stability of plant communities, which could potentially be a factor enhancing ecosystem resilience by buffering plant assemblage changes (De Witte and Stöcklin, 2010; Klimešová et al., 2021). In the high-elevation Mediterranean Apennine mountains, the structure of the clonal forest stand has not been identified. As a result, its role in shaping the natural assemblage of understory herbaceous species is poorly understood. More importantly, the association between the structure and habit of trees and understory communities has not been widely discussed with regard to clonal tree forests. In this context, *F. sylvatica* represents an excellent system within which to investigate the association between tree clonality and understory herbaceous species.

Within the framework of shifting range margins among global species (Pauli et al., 2012), fine-scale near-ground climatic differences may potentially mitigate this shift (Lenoir et al., 2017). Accordingly, in harsh environments, it is hypothesized that cold-adapted species may survive and persist in small refugia to counteract warmer climate trends (Birks, 2015; Hylander et al., 2015). These microrefugia are sheltered microsites climatically decoupled from the meso- and macro-climate conditions (Dobrowski, 2011), allowing for the persistence of endemic and specialized species at their distribution range margins (Keppel et al., 2012). Species community assemblages inhabiting microrefugia have the potential to host a unique and diverse genetic pool within the species (Abeli et al., 2018). Thus, their identification has significant implications for conservation planning in the context of ongoing climate and land-use changes (Selwood et al., 2019).

In this study, we selected the southernmost subalpine Mediterranean beech forest, representing a refuge area that hosts a plant community specific to the topoclimatic context in which it has persisted in the post-glacial period with important implications for conservation. To address the predominant factors that drive changes in stand structure, tree growth habit, and understory vegetation pool of the southern Apennines *F. sylvatica* forest, we selected an elevation gradient spanning from 1,650 to 2,141 m a.s.l. Our specific objectives were to document the elevational variation of (i) forest stand structure, (ii) growth habit of trees from the mountain belt up to the subalpine species range elevation limit, and (iii) their effects on shaping the understory plant community assemblage along with the gradient. We hypothesized that anthropogenic disturbances at low elevations and environmental constraints at high elevations have played a key role in shaping the local spatial structure and growth habit of beech forests and their understory-associated species communities.

## Materials and methods

### Study sites

**Figure 1:**
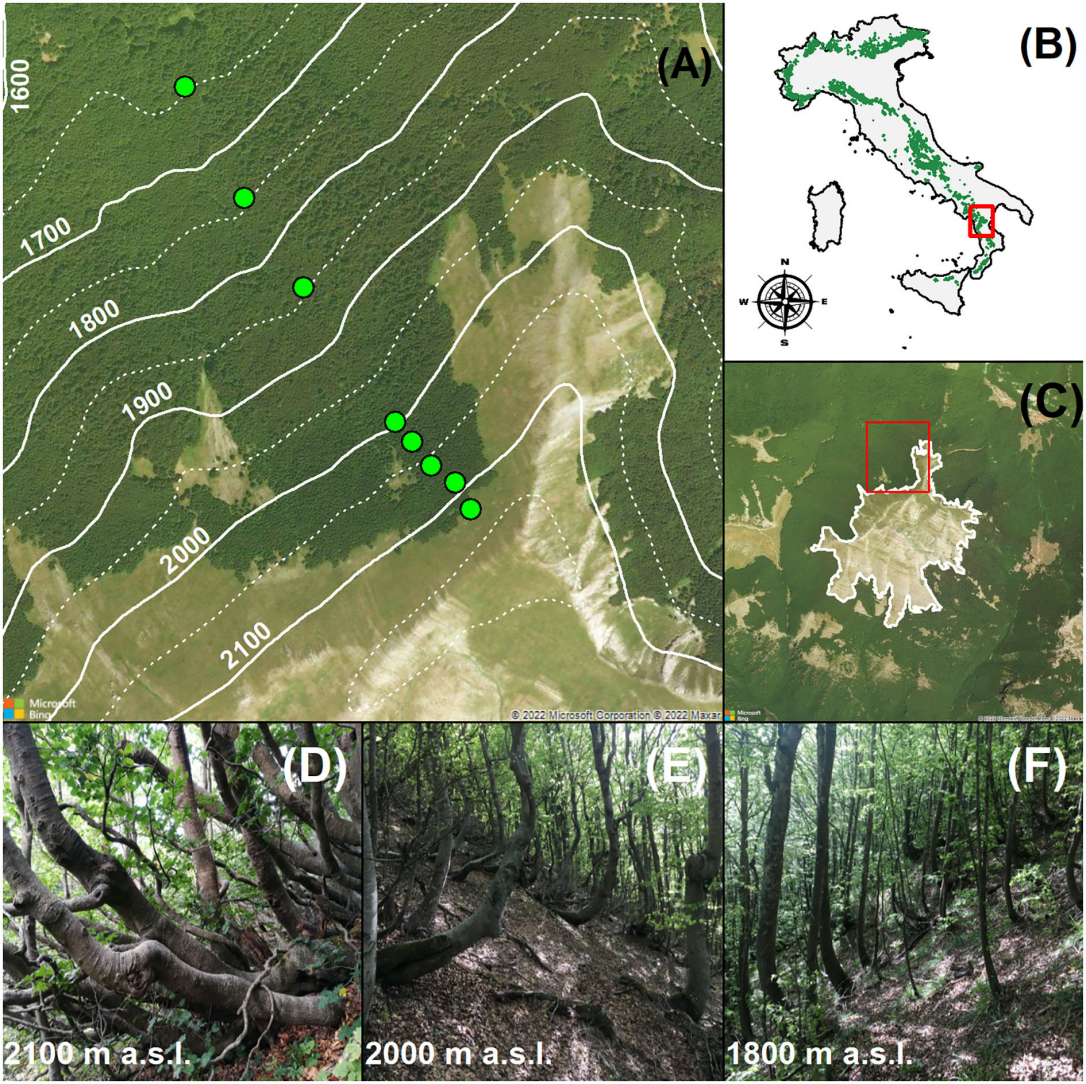
High-elevation monospecific *Fagus sylvatica* forests in the Serra del Prete study area (southern Apennines, Italy). **(A)** Shows the north-western sampled slope of the mountain; the solid (100 m) and dotted (50 m) white contour lines represent elevation intervals; the green circular markers indicate the location of the permanent sampling plots (*n* = 8). In **(B)**, the Alps and Apennines distribution range of *F. sylvatica* in Italy (IV level of Corine Land Cover) is indicated. In **(C)**, white lines denote the upper abrupt and “Krummholz” form (*sensu* Harsch and Bader 2011), and red squares represent the spatial position of the north-western sampled slope of the Serra del Prete mountain. **(D–F)** Show the stand structure of *F. sylvatica* at 2,100, 2,000, and 1,800 m a.s.l., respectively. The aerial images presented in this figure were obtained from Bing Maps (Microsoft^®^ Bing^TM^ Maps API Platform).

The study was conducted on the north-west facing slopes of the Serra del Prete mountain (39°54' N, 16°08' E; peak elevation: 2,181 m a.s.l., southern Apennines), from 1,650 to 2,130 m a.s.l., near the southern limit of *Fagus sylvatica* L. distribution (Figures 1A,B). The forest soil originates from fissured grayish limestone as Haplic Calcisol (FAO Soil Classification System). The climate is characterized by abundant annual precipitation (1,583 mm y^−1^ at 973 m a.s.l.), mainly concentrated from the autumn to the spring (92.4%), with the minimum in summer (7.6%). From mid-November to mid-April, precipitation frequently falls as snow and above 1,900–2,000 m a.s.l., snowpack generally persists up to mid-May. The beech forest above 1,500 m a.s.l. is classified as *a Ranunculo brutii-Fagetum sylvaticae* plant association (*Geranio versicoloris-Fagion sylvaticae* alliance), referred to by the priority habitat code ^*^9210 “Apennine beech forests with *Taxus* and *Ilex*” (Habitat Directive 92/43/EEC, Annex I).

The *F. sylvatica* boundary of the upper elevation limit varied locally according to flank aspects and past land use (Figure 1C). From a biogeographic perspective, the upper elevation limit of *F. sylvatica* is part of the subalpine belt, an ecotone with dwarfed trees and subalpine meadows extending from the treeless belt above to the forested belt below (Löve, 1970). On the southern and eastern slopes, it alternatively appears as abrupt (i.e., with no decreasing tree height and stunted trees) and diffuse forms, ranging from 1,698 to 2,048 m a.s.l., whereas on the northern and western slopes, the upper elevation limit appears alternatively in the abrupt and Krummholz forms (e.g., polycormic and dwarfed trees) spanning from 1,813 to 2,141 m a.s.l. On these slopes, monospecific *F. sylvatica* stands growing below 2,000 m a.s.l. were managed until 1960 for charcoal production. The relict of charcoal kilns is evident up to 1,900 m. Here, the forest stand is structurally mono-layered and dominated by upright and single-stem trees. Above 2,000 m a.s.l., the layering of the multi-stemmed clonal stand was visually verified by excavating the soil and organic layer at the base of the procumbent stems (Supplementary Figure 1).

### Sampling criteria

In summer 2019, eight permanent sampling plots were positioned along an elevational gradient of the north-facing slope of the Serra del Prete mountain (Figure 1A) from a minimum elevation of 1,650 to a maximum elevation of 2,130 m a.s.l. (Table 1), approximately 10 m below the highest upper elevation limit of 2,141 m a.s.l. The plots were established with an elevation distance of approximately 100 m below 2,000 m a.s.l. and ≈40 m above 2,000 m a.s.l. Stand attributes were recorded in rectangular transects or square plots. Their shape and surface area were modulated according to topography constraints and visually estimated stem density (Table 1). Additionally, the WGS84 lat/long coordinates and elevation (m a.s.l.) of the plot center were recorded using the Garmin GPS device (GPSMAP^®^ 66i), while side lengths (m) and mean slope (°) of the plot were measured using the ultrasound hypsometer Vertex (Haglöf^®^ Vertex Laser VL400).

**Table 1:** Geographic coordinates, elevation, layout, and size of sampling plots.

Plot^*^ID	**Coordinates (WGS84)**	**Elevation (m a.s.l.)**	**Slope (°)**	Layout^**^(m × m)	**Area (m^2^)**
1	39°55'35“N 16°09'07”E	1,650	11.78	20 × 50	1,000
2	39°55'29“N 16°09'11”E	1,750	29.20	20 × 50	1,000
3	39°55'25“N 16°09'15”E	1,850	27.62	20 × 50	1,000
4	39°55'18“N 16°09'22”E	2,000	28.48	10.7 × 10.7	114.0
5	39°55'16“N 16°09'23”E	2,040	35.48	7.7 × 7.7	60.0
6	39°55'15“N 16°09'24”E	2,070	33.21	5.5 × 5.5	30.0
7	39°55'14“N 16°09'26”E	2,100	30.04	4.8 × 4.8	23.0
8	39°55'13“N 16°09'27”E	2,130	34.59	3.7 × 3.7	14.0

* In plots 2 and 6, both distance-dependent and distance-independent measurement approaches were applied.

** Rectangular plots, 1–3; square plots, 4–8.

### Stand structure assessment

Both distance-dependent and distance-independent measurement approaches were applied (Pommerening, 2002; Gadow et al., 2012). A distance-independent approach was used to quantify the stand structure attributes in all plots from 1,650 to 2,130 m a.s.l. This approach is known as an area-based approach and is traditionally used for the characterization of forest ecosystems (Gadow et al., 2012). Additionally, in two plots at 1,750 and 2,070 m a.s.l., the distance-dependent approach was applied to identify differences in spatial stand structure organization (Table 1).

#### Distance-independent measurements

According to the distance-independent approach, tree biometric attributes were established in the field survey and scaled at the stand level (Pretzsch, 2009). In each plot, a set of attributes was recorded: tree species, stem number, diameter at 1.30 m (cm) (two orthogonal measurements to account for the irregularity of the stem cross-section), total tree height (m), crown base height (CBH; m) (corresponding to the height of the lowest live branches), and basal stem length (m) (i.e., the length of the stem from the base to a vertical distance from the ground of 2 m). These attributes were used to assess the following array of stand-level attributes: stem density (n ha^−1^), stem number per stool (n. stool^−1^), total tree and crown-base height (m) of dominant trees (the mean height of trees having a height ≥ 75% of the maximum height), dominant foliated tree crown length (m) (the arithmetic differences between total tree height and CBH of the dominant tree), basal area (m^2^ ha^−1^), and quadratic mean diameter (cm) (representing the stem with the mean basal area). Stem diameter and tree height attributes were measured using a caliper (Haglöf^®^ Mantax Blue) and the ultrasound hypsometer Vertex.

#### Distance-dependent measurements

According to distance-dependent measurements, in two selected plots (1,750 and 2,070 m, mono-stem and multi-stem, respectively), the X_i_ and Y_i_ Cartesian coordinates (m) of each tree were measured (Pommerening, 2002) using ultrasound hypsometer Vertex, in addition to the biometric attributes.

### Stand height curve estimation

The stand height curve was used to assess variations in stand structure along with the altitudinal gradient. In each plot, the following semi-logarithmic stand height curve (Pretzsch, 2009) was used to model the relationship between tree diameter at breast height (DBH) and total height (Ht):


(1) $$\begin{array}{l} Ht\;=\;a\;+\;b\;•\;ln\;\left(DbH\right) \end{array}$$

where parameters *a* and *b* represent the intercept and slope of the equation model, respectively, and *ln* denotes the natural logarithm.

### Estimation of plant area index (PAI)

The PAI was measured using an LAI-2000 Plant Canopy Analyzer (PCA; Li-Cor, Lincoln, NE, USA) optical sensor in eight permanent plots. In all plots, the cycle of measurement consisted of six light readings: two references above the canopy and four below the canopy. The two reference readings were collected in an open area near each target plot at the beginning and end of each cycle. All light transmittance readings were acquired by covering the fish-eye lens with a 180° cap-view. The below canopy readings were collected 0.5 m above the ground surface. All measurements were carried out between late July and early August 2018 and 2019, when the canopy leaf areas of *F. sylvatica* stands were at their seasonal maximum, with leaves completely expanded depending on elevation. To avoid the effects derived from the changes in sky conditions between above and below canopy readings, all cycles of measurements were made on cloudless days, at sunset under diffuse light conditions.

### Estimation of stem-shape index

To estimate the degree of vertical alignment of *F. sylvatica* tree stems along with the altitudinal gradient, in the first two basal meters, fixed straight-line/vertical distance from the ground (h_f_), stem length (l_s_), was measured (Jónsson, 2004). In each sampling plot, stem length was randomly measured on a variable number of stems (from 5 to 15) with vertical height >2 m using a metric wheel and a telescopic pole to measure the 2-m fixed vertical distance. Therefore, the stem form index (SFI_i_) was computed as the ratio between stem length (l_s_, m) and 2-m fixed vertical height (h_f_, m) as follows:


(2) $$\begin{array}{l} SFI_{i}=\frac{l_{s}}{h_{f}} \end{array}$$

The stem form index represents a dimensionless quantity that assumes values ≥1. When SFI_i_ = 1, the stem length and fixed 2-m vertical height were equal and the stem was totally upright, whereas when SFI_i_ > 1, the stem length was longer than 2 m. When SFI_i_ >> 1, the stem was aligned downhill for many meters before curving up into a vertical position. In these two cases, the stem appeared to be bent and dwarfed. Therefore, the stem form index represents a tree growth-form functional trait attribute that describes the degree of departure of the basal portion of the stem from the vertical alignment.

### Stem age determination

A cluster of ramets layered from the same genets and growing at 2,040 m a.s.l. was sampled to determine the age structure of the *F. sylvatica* clonal stem. All cores were taken 15–45 cm from the base of the stem using an increment borer. To visualize the tree ring boundary, cores were mounted on wooden strips, flattened with a stainless-steel surgical blade, and mechanically sanded by a series of progressively finer-graded sandpaper until the xylem cellular structure was clearly visible under a 6.4–40× magnification binocular lens (Leica, Germany). White chalk was also used to enhance the contrast between the anatomical elements. Annual rings of each core were manually counted and visually cross-dated, paying special attention to avoid counting false rings.

### Understory vegetation analysis

All vascular plant species were recorded for each plot (Table 1), and their coverage abundance was estimated according to the Braun-Blanquett method for each layer (tree, shrub, and herb). The nomenclature and chorological type of the species follow the checklist of Italian flora and Flora of Italy, respectively. A community matrix of eight surveys × 61 species was obtained. A complete plant list with species cover abundance is provided in Supplementary Table 1. Given the biogeographic peculiarity of the microthermic beech forests at its southern latitudinal limit range in Europe, we selected the herbaceous species of biogeographic interests in the understory layer (Supplementary Table 2). These species include southern Apennines beech forest target species (Biondi et al., 2014), which are narrow-range fidelity species, limited to South Apennines or with an extended range from South Apennines to the South Balkan region (Di Pietro, 2009; Willner et al., 2009; Biondi et al., 2014). In addition, orophytes and Apennines are endemic, subendemic, and boreal species at the southern limit of their distribution, which are rare or uncommon in South Apennines' beech forests that include generalist forest species. The literature used for the selection of species of biogeographic interests is listed in Supplementary Table 2.

### Climatic data

To assess the thermic climate experienced by *F. sylvatica* at the subalpine belt during the growing season, daily air temperature data measured 2 m above ground from 2006 to 2021 were obtained from the closest weather station (Campotenese, 39°.87' N 16°.06' E, 973 m a.s.l.) and scaled to the Serra del Prete study site. Taking the elevation differences between the weather station and the study site into account, the daily mean temperatures of the growing season were scaled to 2,000 m (upright elevation tree limit) and 2,140 m (upper elevation species range limit) assuming a common altitudinal lapse rate of 0.006 K m^−1^ (Barry, 2008). At 2,130 m, the average length of the growing season from the year 2017 to 2020 accounted for 162 (±10) days, approximately spanning from early May to middle October. Length of the growing season data was extracted from the pan-European High-Resolution Vegetation Phenology and Productivity (HR-VPP) product suite available from Copernicus Land Monitoring Services (https://land.copernicus.eu/pan-european/biophysical-parameters/high-resolution-vegetation-phenology-and-productivity).

### Data analysis

A non-linear regression model was used to determine the relationship between the DBH and the total height of the trees. The parameters of the semi-logarithmic model were estimated using the Gauss-Newton non-linear least squares method (Zar, 2010). The starting values for the parameters were estimated using the ordinary least squares linear regression model. Efron's pseudo R-squared (R^2^) and root mean square error (RMSE) were used to evaluate the goodness of fit of the non-linear regression model. Analysis and management of the biometric data were performed in R using the package “nlstool” (Baty et al., 2015).

A standardized univariate L-function spatial pattern analysis was applied to determine the type of spatial aggregation of trees (Baddeley et al., 2014). The analysis was performed by comparing the two sampled stands growing at 1,750 and 2,070 m a.s.l., respectively. The significance of deviation from the null hypothesis of complete spatial randomness (CRS) was assessed by implementing a Monte Carlo simulation with several simulated point patterns of 39 random values. The upper and lower envelopes were computed pointwise for each tree-to-tree distance. Such pointwise envelopes represent critical points for the Monte Carlo test at a significance level of α = 0.05. Therefore, based on the score of the estimated L-function scores, three types of stem spatial aggregation can be discriminated, i.e., clumped, random, or hyperdispersed, if the L-function score is significantly higher, equal, or lower than the pointwise envelopes, respectively. Analysis and management of the spatial data were performed in R using the package “spatstat” (Baddeley and Turner, 2005).

To assess the effect of the *F. sylvatica* canopy cover in shaping herbaceous community assemblages, the relationship between stand structure and narrow-range species composition along with the altitudinal gradient was investigated using principal component analysis (PCA). Both stand structure metrics and species percentage abundance values were square root transformed to simultaneously assure multivariate normality of variable distributions and independence between components of the PCA (Henderson, 2003). The contribution of variables to the explanation of data variability was assessed using correlation coefficients with both principal components. PCA was performed in R using the “FactoMineR” package (Lê et al., 2008).

## Results

### Stand structure

Density-related stand structure attributes increased with elevation, while the mean tree size progressively decreased (Table 2). In the single-stem physiognomy, density increased progressively up to ≈6,200 stem ha^−1^ at 2,000 m, while in the multi-stem physiognomy, it increased progressively up to ≈71,000 stem ha^−1^ at 2,130 m, and the basal area and quadratic mean diameter increased and decreased with elevation, respectively (Table 2).

**Table 2:** *F. sylvatica* stand structure attributes were sampled along with the elevation gradient of the north-western slope of Serra del Prete mountain.

**Elevation (m a.s.l.)**	**Growth habit**	**Stem density (n ha^−1^)**	**Stand basal area (m^2^ ha^−1^)**	**Quadratic mean diameter (cm)**
1,650	Single-stemmed	1,670	47.6	19.0
1,750		1,360	39.7	19.3
1,850		1,780	49.2	18.8
2,000		6,174	56.9	11.0
2,040	Multi-stemmed	7,325	51.0	9.4
2,070		13,900	75.4	10.3
2,100		11,651	78.2	9.4
2,130		70,729	67.9	3.5

**Figure 2:**
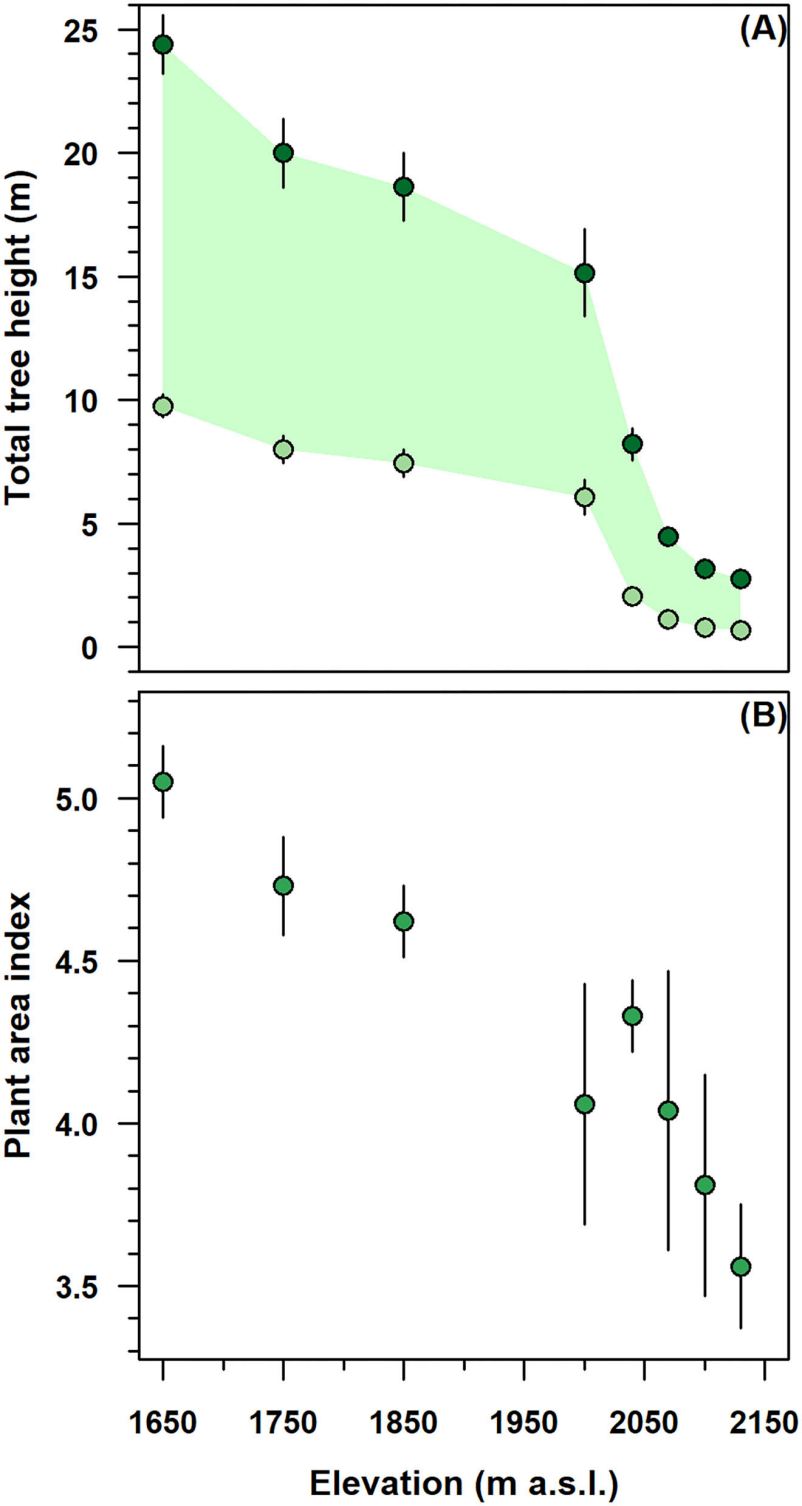
Elevation variation of height-related attributes and plant area index (PAI) in the single-stemmed (1,650–2,000 m) and multi-stemmed (2,040–2,130 m) *F. sylvatica* stands of the north-western slope of Serra del Prete mountain. **(A)** Dark green and light green circles denote total and live crown base tree heights, respectively, while the green band represents the live foliated crown length. **(B)** Green circles represent PAI. In both panels, vertical bars indicate first standard deviation (SD).

The height-related attributes and the PAI of stands decreased considerably along with the altitudinal gradient (Figure 2). Below 2,000 m, in the monostem stand, the total tree and CBH were gradually increased from 15 to 25 and from 7 to 10 m, respectively (Figure 2A). Above this elevation, multi-stemmed tree height parameters were sharply decreased to 3 m in total height and 1 m in CBH; meanwhile, their variability substantially reduced to 0.5–1 m.

In mono-stem stands, from 1,650 to 2,000 m, the decrease in average total height was higher (≈33%) than the decrease (≈22%) in live CBH (Figure 2A). The *F. sylvatica* crowns were ~10–15 m long and 7–10 m far from the ground surface. In contrast, above 2,000 m, the crown length was sharply decreased from 10 to 3 m near the species range elevation limit. In the multi-stemmed stand, the live CBH progressively approached the ground and was 1–2 m away from the soil surface.

In the mono-stem structure, from 1,650 to 2,000 m, the PAI was decreased by 30.7%, with values exhibiting very low variability (Figure 2A). In the multi-stemmed structure, with an increasing elevation from 2,000 to 2,130 m, the PAI decreased to a minimum of 3.6, while its variability increased consistently.

### Stand height and tree growth form

**Figure 3:**
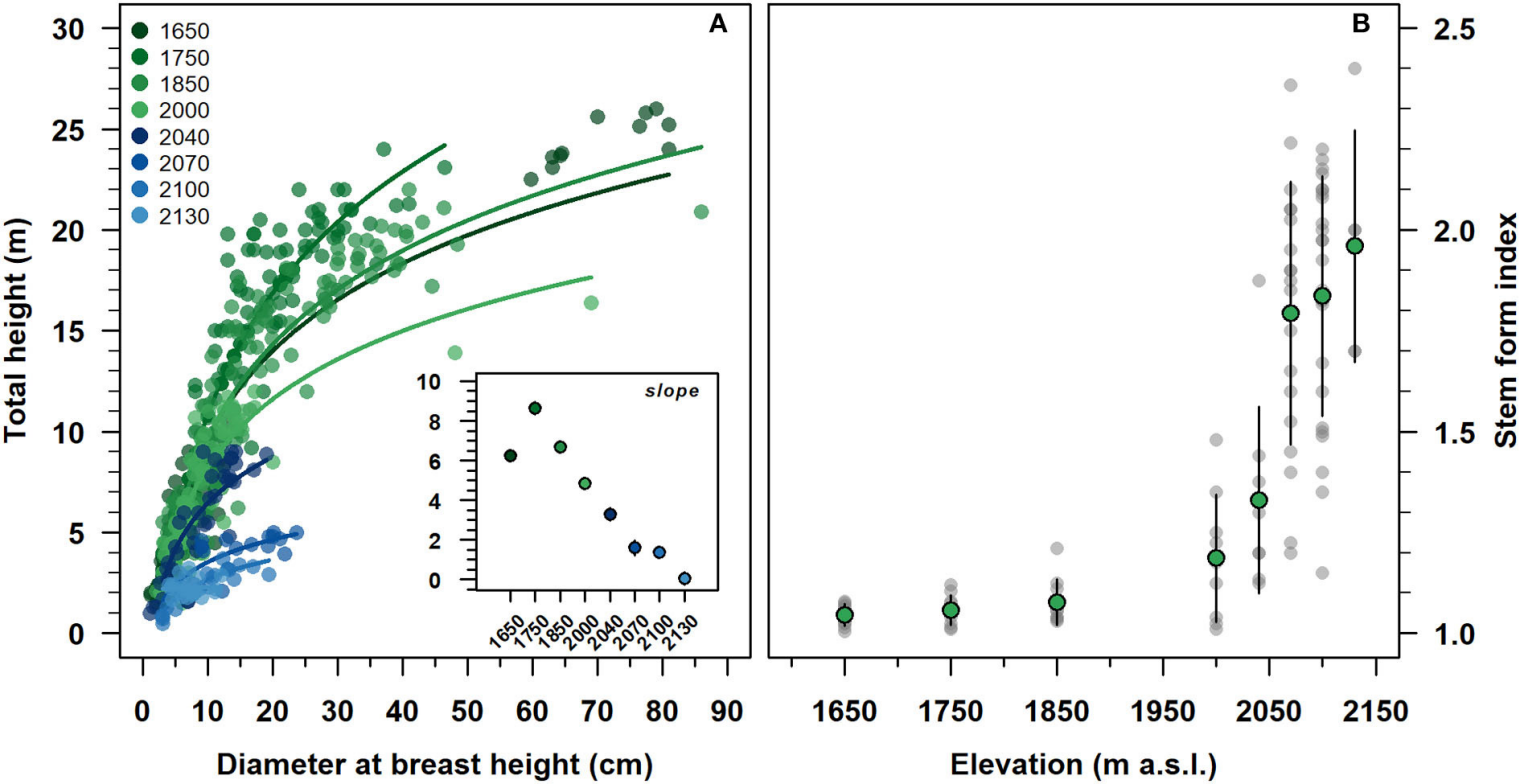
Relationship between diameter at breast height and total tree height **(A)**, and variation of stem form index **(B)** of *F. sylvatica* from high elevation up to the species range elevation limit on the north-western slope of Serra del Prete. **(A)** Lines represent the regressed stand height semi-logarithmic model. The inset plot shows the estimated regression slope parameters of the non-linear regression model. Green and gray circles represent mean values and data observations, respectively. In the inset (see also Supplementary Table 1) and **(B)**, vertical bars indicate first standard deviation (SD).

With an increasing elevation, the stand height curve gradually shifted downward to the origin of the axis (Figure 3A). At the same time, the estimated slope parameters were progressively decreased, reaching a minimum of 2,130 m. All were statistically different from zero, except for the slope parameter of 2,130 (inset Figure 3A and Supplementary Table 1). The intercepts were decreased along with the altitudinal gradient, becoming statistically not significant from 2,040 to 2,100 m. Modeled stand height curves showed an R^2^ range of 0.55–0.90 from 1,650 to 2,100 m and close to zero at 2,130 m (Supplementary Table 1).

The stem form index increased exponentially with increasing elevation (Figure 3B). Below 2,000 m, the average values of the stem shape index were approximately equal to 1.12 and with low variability. Above 2,000 m, both the mean shape index and its variability were steeply increased from 1.25 to 2.02, with increasing of elevation.

### Stand spatial structure

**Figure 4:**
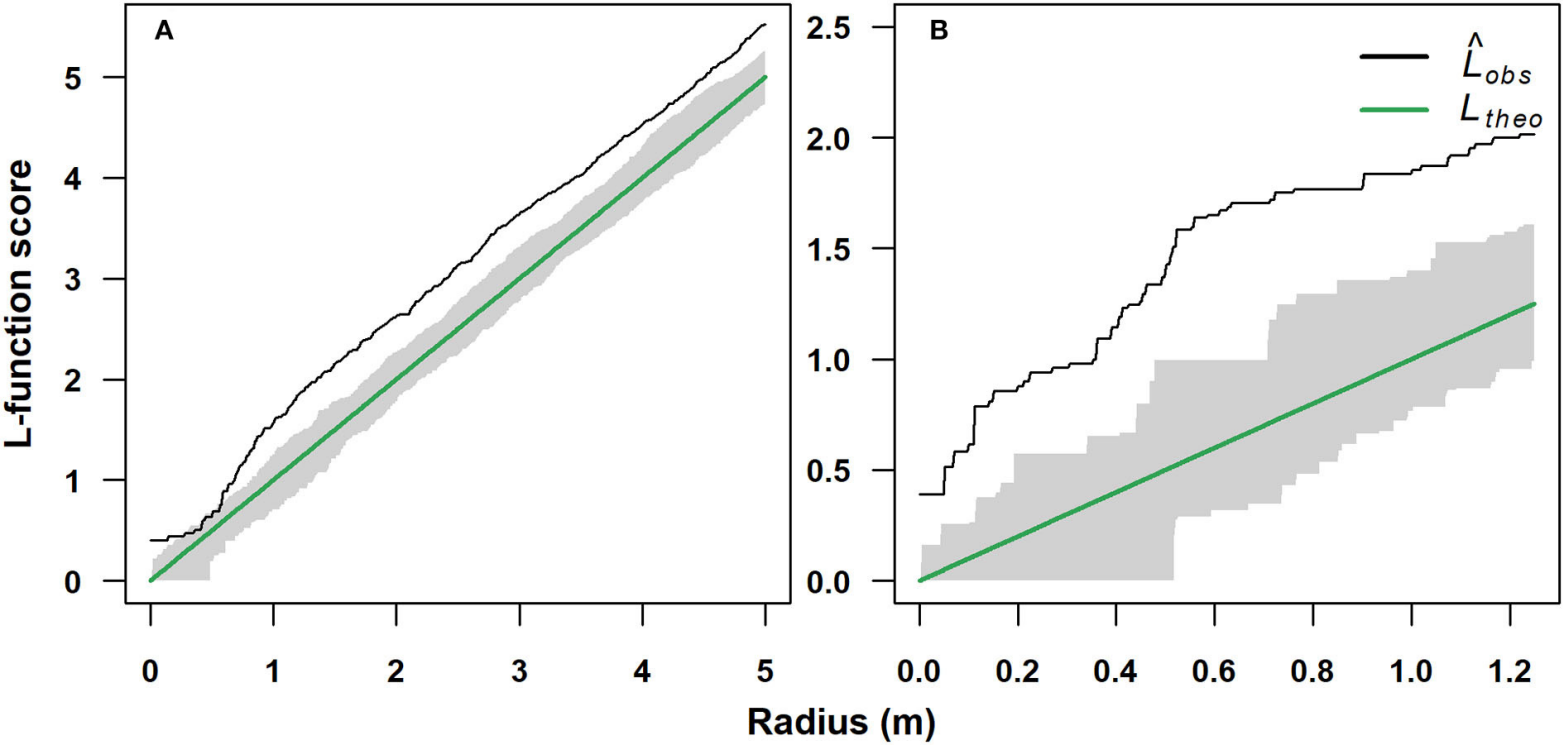
Standardized univariate L-function spatial pattern analysis of a single-stem and a multi-stem *F. sylvatica* stands growing at two different elevations on the north-western slope of Serra del Prete: **(A)** 1,750 m (mono-stem) and **(B)** 2,070 m (multi-stem). The solid black line represents the observed empirical L-function (L_obs_), while the solid green line represents the theoretical L-function for Poisson random spatial distribution (L_theo_). The gray bands represent the 95% acceptance confidence interval (CI) under the hypothesis of complete spatial randomness.

The spatial distribution in the single-stem (1,750 m) and multi-stem (2,070 m) stands was significantly clumped (*p* < 0.05). The single-stem stand showed a random spatial distribution for a short range of inter-stem distance of 0.3–0.7 m (Figure 4A), while the multi-stem stand showed a clumped spatial assemblage for each inter-stem distance (Figure 4B).

### Age structure in a polycormic unit (Nest)

The age-class structure of a polycormic unit growing at 2,040 m showed that the clonal stem population is uneven-aged, mainly established in the last 150 years with a high frequency of <80 years (Supplementary Figure 2). The age span ranged from the oldest stem of 240 years (established in 1778) to the youngest with an age of 34 years (established in 1984).

### Herbaceous species of biogeographic interests

**Figure 5:**
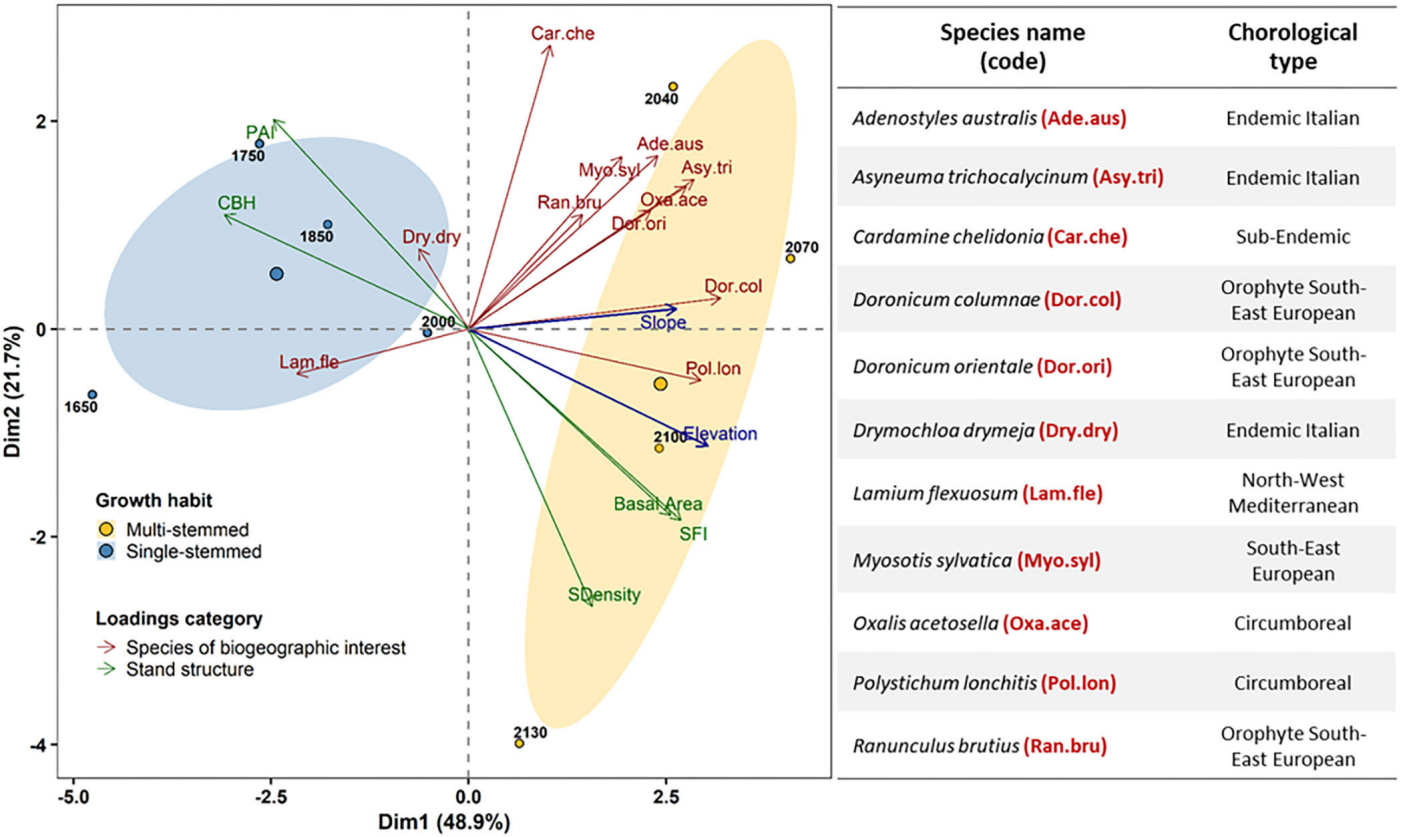
Relationship among abundance of biogeographic interests target herbaceous species and topographic and structural attributes of *Fagus sylvatica* stands covering the north-western slope of the Serra del Prete mountain. Circular points represent plane coordinates of altitudinal sampling plots according to growth habit (supplementary qualitative category): yellow for multi-stemmed and black for single-stemmed. Arrows represent loadings (variables), colored according to the category of forest canopy attributes: red for target species abundance and green for stand structure metrics. Dark blue arrows represent continuous supplementary topographic variables (elevation and slope). Ellipses are drawn around the coordinates of the centroid of each growth habit with a 95% confidence (CI) band. Stand structure metric: CBH, live crown base height; PAI, plant area index; basal area, stand basal area; SDensity, stem density; SFI, stem form index. The table on the left includes the scientific name, code, and chorological type for each herbaceous species of biogeographic interest.

Overall, 70.6% of the variance in species assemblage and topographic and structural attributes of *F. sylvatica* stands was explained by the first and second PCA dimensions (Figure 5). The first component expresses more than double the variability explained by the second dimension. Six species of biogeographic interests were significantly and positively correlated with the first component: *Doronicum columnae* (0.94, *p* < 0.001), *Polystichum lonchitis* (0.87, *p* < 0.01), *Asyneuma trichocalycinum* (0.84, *p* < 0.05), *Oxalis acetosella* (0.82, *p* < 0.05), and *Adenostyles australis* (0.71, *p* < 0.05).

Among the stand structure attributes that were significantly correlated with the first component, the stem form index (0.80, *p* < 0.05), and stand basal area were positively correlated (0.76, *p* < 0.05), whereas CBH (−0.91, *p* < 0.001) and PAI (−0.73, *p* < 0.05) were both negatively correlated. *Cardamine chelidonia* (0.81, *p* < 0.05) was positively correlated with the second component, whereas stem density was negatively correlated (−0.79, *p* < 0.05). Continuous supplementary variables' elevation (0.89, *p* < 0.01) and slope (0.78, *p* < 0.05) were both positively correlated with the first PCA dimension. The qualitative supplementary variable describing the stand growth habit results was correlated positively with the first component (0.76, *p* < 0.01). Moreover, in the first dimension, the multi-stemmed growth habit (2.69, *p* < 0.01) had a positive coordinate, whereas the single-stemmed tree growth habit had a negative coordinate (−2.69, *p* < 0.01).

### Subalpine belt thermic climate

At the current *F. sylvatica* upright tree limit elevation (2,000 m), the adjusted isotherm was placed at 9.6°C seasonal mean temperature. Moving to the upper elevation range limit of 2,140 m, *F. sylvatica* occurs as Krummholz and is placed at an adjusted isotherm of 8.7°C.

## Discussion

In the subalpine belt, the growth habit of *F. sylvatica* trees changes from upright and progressively basal-curved single-stemmed to polycormic procumbent clonal stems, forming a canopy cover with a live CBH that is progressively closer to the ground and 1–5 m away from the genet founder. All these physiognomic changes are a common spatial pattern above a topographical threshold of 2,000 m a.s.l., where the reiteration potential of *F. sylvatica* through layering reflects the combination of the microclimate benefit of being small in stature and dense in the canopy and the repeated damage by mechanical snow pressure that prevents upright tree growth. The main physiognomic structural changes observed in this unicum of beech stand are first represented by the progressive gravitropic form response of the single stem to external mechanical stimuli, which produce the basal stem curvature (Groover, 2016) and then by a multi-stem stand (Harsch and Bader, 2011). In general, the reiteration of above-ground biomass by coppicing is discordant with the undisturbed clonal reproductive strategy of the species observed at high elevations, where the growth form and mortality are affected by stochastic (e.g., mechanical failure affected by disturbances and snow pressure) rather than deterministic processes fueled by self-thinning.

Such a distinctive clonal propagation strategy by layering represents a common functional trait documented in many other broadleaved and conifer species growing at their elevation and latitude limits (Holtmeier, 2009; Öberg and Kullman, 2011). Although the clonal layering structure of *F. sylvatica* was recognized in Central Europe (Vacek and Hejcman, 2012), it represents the first record in the southernmost *F. sylvatica* Mediterranean distribution range. The multi-stemmed structure by layering could well represent a phenotypic plastic response of *F. sylvatica* to environmental constraints rather than the result of basal resprout after human disturbance by coppicing. This clonal growth habit could also play a crucial role in shaping and preserving understory community species linked to *F. sylvatica* glacial refugia. Moving to this multi-stemmed subalpine belt, the canopy cover buffers mean near-ground extreme temperatures in the growing season of 1.0°C as compared to open-field near-ground air temperatures, as recently demonstrated by Rita et al. (2021) in the same *F. sylvatica* Krummholz site. This microclimatic buffering indicates temperature non-limiting for tissue formation and differentiation processes and thereby has the potential to offset climate warming at the local scale, reducing the disequilibrium between ecosystem responses and anthropogenic climate change. This suggests that in the studied Mediterranean subalpine belt, the clonal regeneration of *F. sylvatica* could be involved in the formation of thermal niches suitable for the persistence of specialized understorey species.

### Stand structure and growth habit

The genesis of such a multi-stemmed structure is well-supported by previous empirical findings on *F. sylvatica* advancing species range limit of the Apennines (Bonanomi et al., 2021). Here, the growth habit of isolated *F. sylvatica* founder outposts (genets) of clonal groups (ramets) is very distinctive, showing a dense group of plagiotropic branches at the base (skirt), followed by successful acropetally in a portion of the stem without branches and a small asymmetric crown in the distal part. The meristems of the basal branch bank, ontogenetically young, represent the “cone of juvenility” (Preece, 2008), prone to produce adventitious roots as an epigenetic acclimation (*sensu* Douhovnikoff and Dodd, 2015) to the mechanical disturbance of the snowpack. The basal branches are decoupled from the surrounding atmosphere and have a higher aerodynamic resistance and tissue-to-air temperature differentials than crown branches (Wilson et al., 1987). This aerodynamic decoupling could enhance photosynthate production invested primarily in the close below-ground biomass before the emergence of orthotropic shoots (Preece, 2008). Although the proliferation of layered clonal stems is an energy-demanding process, it has the potential to improve both water and nutrient uptake by an amplified root system (Šenfeldr et al., 2016) and thus improves species persistence in harsh subalpine environments.

Therefore, the layering of *F. sylvatica* basal lateral branches in the uppermost soil layers can produce adventitious roots and new autonomous secondary stems (ramets), forming dense clonal populations that progress downward on the steepest slopes as a consequence of snowpack load. In the multi-stem physiognomy of subalpine belts (above 2,000 m), the stems mechanically solicited by snow load and gravitropism respond thigmomorphogenetically by retarding their elongation growth (Moulia and Fournier, 2009) and expand mainly subhorizontally. Both the increase in the stem form index and stem density observed above 2,000 m could be related to the snowpack pressure experienced by trees during the winter season (Supplementary Figure 3) contributing to its Krummholz structure, as observed in other tree species across biomes (Homma, 1997; Maher et al., 2020).

Tree height reduction along with elevation is parallel to a reduction in mean stem diameter, suggesting that approaching the uppermost elevation limit, the primary growth and secondary growth of *F. sylvatica* are temperature-limited rather than nutrient-limited (Rossi et al., 2016; Mayor et al., 2017; Huang et al., 2020). The progressive reduction of the tree height-diameter relationship (see inset in Figure 3A) along with the altitudinal gradient reflects a more marked decrease in tree height variation than in stem diameter. As a result, when approaching the uppermost elevation limit, the allometry of clonal stems switches disproportionately, suggesting a trade-off for the competition of resources from a vertical to a horizontal plane, indicating that the profile of clonal stems of *F. sylvatica* increases taper nearing its upper elevation limit (Körner, 2012a).

Above the *F. sylvatica* upright tree limit (2,000 m), seasonal mean temperature results are not restrictive to physiological growth processes. At the upper elevation limit of 2,140 m, air temperature is 2.3 K higher than the mean treeline seasonal air temperature of 6.4°C, and the growing season amounts to 162 days, compared to the minimum of 90 days reported for the global climatic treeline (Körner, 2012b). There is a large body of evidence that growth activity in cambial and apical meristems becomes negligible below 5°C temperature (Rossi et al., 2008). Therefore, the temperatures at the current *F. sylvatica* upper range do not limit growth processes. However, the microclimate benefit of being small in stature and dense in canopy allows *F. sylvatica* to grow at higher elevations than upright standing trees.

In the subalpine belt, canopy cover exhibits a progressive reduction in the PAI, with values (PAI ≈ 3) comparable to those of dwarfed tree communities growing under the same bioclimatic conditions (Körner, 2012a). Due to the high stem density and the short inter-distance between stems, it is plausible that woody organs contribute considerably to the PAI. Since leaf area is allometrically related to biomass (Bartelink, 1997), the observed lower PAI values in the multi-stemmed stand could reflect a disproportional allocation of resources between aboveground woody organs than leaf biomass. In contrast to the PAI, the stand basal area progressively increases with elevation, showing values comparable with those found in *Nothofagus* Krummholz stands edge in the Southern Hemisphere (Cullen et al., 2001).

### Spatial aggregation, demography, and ecosystem engineering

The spatial aggregation in the single-stemmed stands is the legacy of the use of previous coppice systems in charcoal production and their subsequent conversion into a high forest starting from the 1950s. By contrast, multi-stemmed stands are the result of a natural layering process. Clonal recruitment documented only at 2,040 m suggests an asynchronous process producing an even-aged clonal stem population. Moreover, the maximum age of 240 years could be approximate to the age of the ancestor, although the precise identification of the rot stem genet remains uncertain. In this subalpine clonal beech forest, seed regeneration could be temporally and spatially discontinuous and was observed only at 2,070 m a.s.l. as sapling cohorts were spatially restricted within the canopy gaps (Supplementary Figure 2).

Having such a physiognomy, *F. sylvatica* clonal trees interact with the surrounding physical environment through the process of “ecosystem engineering” (Jones et al., 1994) by altering the local microclimate. For instance, the polycormic growth habit trap and holding snow against glide forces increase the depth of the snowpack and its persistence for 4–5 months (Rita et al., 2021). Therefore, crown branches buried in the snowpack remain thermally insulated and buffered against the local macroclimate, while the top crown fraction above the snow may be subjected to the abrasive force of ice particles driven by winds (Supplementary Figure 3). Recently, Hagedorn et al. (2014) found that increasing treeline tree growth can be linked to increased snowfall precipitation as a consequence of higher winter precipitation. If the snowpack upregulated the shape and size of our Krummholz structure, then the negative trend of winter precipitation documented in the last century in the Pollino Massif, mainly in January (Brunetti et al., 2012), could have altered the multi-stemmed structure and its spring phenology linked to the temporal and spatial dynamics of snowpack melting.

### Understory assemblage

Such a distinctive canopy layer documented in the multi-stemmed subalpine belt may consist of a stable habitat and ecological niches for different herbaceous taxa of biogeographical interest. Abiotic and biotic parameters, coupled with canopy-mediated microclimate observed near treelines (Rita et al., 2021), may act as effective ecological filters to preserve and conserve a unique herb species assemblage in the refugia from thermophilization. In particular, a set of perennial clonal forbs and ferns, such as *D. columnae* and *P. lonchitis*, are favored in sites with stand-replacing disturbances (Franklin et al., 2020), such as snow avalanches, whereas specialist forest species, such as *A. trichocalycinum* and *O. acetosella*, are characterized by low dispersal ability (Willner et al., 2009), persist in favorable ecological niches. In the assemblage of the herbaceous species, we also found abundant narrow species, such as *A. trichocalycinum* and *C. chelidonia*, which are endemic and subendemic to microthermal beech forests in the southern Apennines (Di Pietro, 2009; Willner et al., 2009). Additionally, we found rare orophytes and boreal and Apennines endemic species at their southern limit of distribution, under conditions of climate and topographical habitat limitation, such as *D. columnae, P. lonchitis, A. australis*, and *O. acetosella. D. columnae* is a south-eastern orophyte species and shady limestone rock generalist forest species linked to the Apennines and Balkan connection during the last glaciation and mainly diffuses in the Balkan Peninsula in the subalpine beech forests (Karadžić, 2018). *P. lonchitis* is a circumboreal wintergreen fern typical of long snow-covered alpine and subalpine screes (Biondi et al., 2014) and differential species of subalpine beech forest species in the Alps. *P. lonchitis* is also a relict glacial species, extremely rare in the Apennines, and has not been reported in floristic-vegetational data of beech forests in southern Apennines. *A. australis* is an endemic species that reaches the southern limit of distribution in the study area (Palermo et al., 2002). It is a tall herb and generalist species that mainly occupies the margin of the microthermic beech forests from the upper mountain to the subalpine belt under wet edaphic conditions (Biondi et al., 2014). *O. acetosella* is a circumboreal hygromorphic species that is specialist of beech forests and is limited to deep shade and humid habitats (Packham, 1978). It is important to note that in *F. sylvatica* forests along with the Apennines, phytosociological community surveys were absent above 2,000 m, and the only one was referred to our study site. Excluding the Pollino Massif (where falls our study site) and the insular Etna volcano, all mountains in the southern Apennines are <2,000 m (Fredi and Lupia Palmieri, 2017). For several species (e.g., *Ranunculus brutius* and *A. trichocalycinum*), altitudinal limits expand in the subalpine belt up to 2,140 m. The southern Apennines have been generally recognized as glacial refugia for *F. sylvatica* and the associated understory species community (Huntley and Birks, 1983). The recorded understory species of biogeographic interest suggest that the remnants and untouched *F. sylvatica* stand could have acted as refugia for narrow endemic species, Apennines endemic, south-eastern orophytes, and circumboreal species through the last glacial period. These elements contribute to the significance of the unique biogeographic pattern found in the edge area of *F. sylvatica* distribution.

## Conclusions

The studied *F. sylvatica* forest represents a unicum in the Mediterranean Basin. It grows at its lower latitudinal distribution in the center of the Mediterranean Basin within the core of the southernmost Apennines glacial refugia, where it spans along with its widest elevation range from 1,000 to 2,141 m a.s.l. Here, the growth habit of *F. sylvatica* trees changes from upright to progressively basal-curved single-stemmed to polycormic procumbent clonal stems, along with an increasing elevation. For the first time in the Mediterranean Basin, we documented the spatial complexity and structure of an unknown relict clonal *F. sylvatica* stand growing under the environmental constraints imposed by low temperature and snowpack in the subalpine belt. Without anthropogenic disturbance, *in situ* persistence by clonal layering, rather than human-induced resprouts, represents a strategy of *F. sylvatica* conducive to the colonization of empty space by growth under accentuated gravitropic and effective in trapping snow. Here, the architecture and climate buffering of the clonal *F. sylvatica* canopy cover allows understory species communities to persist in their fundamental niches over time. The conservative role of this *F. sylvatica* clonal forest as a microrefugia during past, current, and future climatic fluctuation events suggests its inclusion in the priority area networks of the Mediterranean Basin. Therefore, preserving the subalpine *F. sylvatica* clonal stand structure and underlying functions and processes from human activities represents a strategic long-term conservation action of emblematic glacial and climatic refugia.

## Data availability statement

The original contributions presented in the study are included in the article/Supplementary material, further inquiries can be directed to the corresponding author/s.

## Author contributions

LS and AS: conceptualization and methodology. LS: data curation, formal analysis, software, validation, and visualization. LS, AS, AR, VM, MZ, EA, MA, GT, SM, and GB: investigation. AS: resources and supervision. LS, AS, and MA: writing—original draft. LS, AS, MA, AR, MB, EA, MZ, GB, VM, GT, and SM: writing—review and editing. All authors contributed to the article and approved the submitted version.

## Funding

This work was partially supported by the Ph.D. program at the School of Agricultural and Food Sciences granted to VM and MZ in the Department of Agricultural Sciences, University of Naples Federico II. This research did not receive any specific grants from funding agencies in the public, commercial, or not-for-profit sectors. The APC was funded by Fondazione con il Sud – Progetto: Sve(g)liamo la dormiente CUP E78D19000750005, granted to AS.

## Conflict of interest

The authors declare that the research was conducted in the absence of any commercial or financial relationships that could be construed as a potential conflict of interest.

## Publisher's note

All claims expressed in this article are solely those of the authors and do not necessarily represent those of their affiliated organizations, or those of the publisher, the editors and the reviewers. Any product that may be evaluated in this article, or claim that may be made by its manufacturer, is not guaranteed or endorsed by the publisher.
