# Supplementary material for: Clonality drives structural patterns and shapes the community assemblage of the Mediterranean Fagus sylvatica subalpine belt
Source: Front Plant Sci. 2022 Sep 16;13:947166. doi: 10.3389/fpls.2022.947166 (PMC9523587; doi:10.3389/fpls.2022.947166)
Supplement: Supplementary file 1 [file Data_Sheet_1.docx]

Supplementary Material 2

## Supplementary Tables

Supplementary Table S1. Summary of estimated parameters and goodness of fit statistics for stand height semi-logarithmic curves. Parameters a and b represents the intercept and slope of each equation model respectively. Estimate represents the values of the parameters; SE the standard error of the parameter estimates, t and p the Student’s t-value and p-value respectively; RMSE and R2 indicate respectively the root mean square error and the Efron’s pseudo R-squared.

| **Altitude** | **Parameter** | **estimate** | **SE** | ***t*** | ***p*** | **RMSE (m)** | **R^2^** |
| --- | --- | --- | --- | --- | --- | --- | --- |
| 1650 | *a*_1_ | -4.67 | 0.32 | -14.64 | 0.0000 | 1.72 | 0.90 |
|  | *b*_1_ | 6.24 | 0.17 | 36.62 | 0.0000 |  |  |
| 1750 | *a*_2_ | -9.03 | 0.89 | -10.19 | 0.0000 | 1.99 | 0.84 |
|  | *b*_2_ | 8.66 | 0.32 | 26.95 | 0.0000 |  |  |
| 1850 | *a*_3_ | -5.68 | 0.46 | -12.24 | 0.0000 | 1.92 | 0.88 |
|  | *b*_3_ | 6.69 | 0.19 | 35.56 | 0.0000 |  |  |
| 2000 | *a*_4_ | -2.94 | 0.57 | -5.13 | 0.0000 | 1.44 | 0.80 |
|  | *b*_4_ | 4.86 | 0.27 | 18.06 | 0.0000 |  |  |
| 2040 | *a*_5_ | -1.09 | 0.67 | -1.64 | 0.1078 | 1.34 | 0.73 |
|  | *b*_5_ | 3.29 | 0.31 | 10.49 | 0.0000 |  |  |
| 2070 | *a*_6_ | -0.14 | 0.98 | -0.14 | 0.8893 | 0.70 | 0.55 |
|  | *b*_6_ | 1.60 | 0.37 | 4.29 | 0.0006 |  |  |
| 2100 | *a*_7_ | -0.47 | 0.30 | -1.59 | 0.1246 | 0.40 | 0.81 |
|  | *b*_7_ | 1.38 | 0.14 | 9.81 | 0.0000 |  |  |
| 2130 | *a*_8_ | 2.14 | 0.63 | 3.37 | 0.0034 | 0.43 | 0.00 |
|  | *b*_8_ | 0.07 | 0.32 | 0.21 | 0.8372 |  |  |

**Supplementary Table S2**. Literature source list for the selection of understory herbaceous species of biogeographic interests.

| **Species** | **Data source** |
| --- | --- |
| *Adenostyles australis* (Ten.) Iamonico & Pignatti | Palermo A.M., Pellegrino G., Noce M.E.; Bernardo L., Musacchio A., 2002. Patterns of genetic variability in populations of Adenostyles Cass. complex (Asteraceae) along the Apennine chain. Delpinoa 44: 103-114 |
| *Asyneuma trichocalycinum* (Ten.) K. Malý | Gentile S., 1970. Sui faggeti dell’Italia meridionale. Atti Ist. Bot. Univ. Pavia serie 6, 5 (1969): 207-306.  Di Pietro R., Izco J., Blasi C., 2004. Contribution to the nomenclatural knowledge of Fagus sylvatica woodlands of southern Italy. Plant Biosystems 138(1): 27 – 36.  Di Pietro R. 2009. Observations on the beech woodlands of the Apennines (peninsular Italy): an intricate biogeographical and syntaxonomical issue. Lazaroa 30: 89-97.  Willner W, Di Pietro R, Bergmeier E. 2009. Phytogeographical evidence for post-glacial dispersal limitation of European beech forest species. Ecography 32:1011–1018.  Biondi E., Blasi C., Allegrezza M., Anzellotti I., Azzella M.M., Carli E., Casavecchia S., Copiz R., Del Vico E., Facioni L., Galdenzi D., Gasparri R., Lasen C., Pesaresi S., Poldini L., Sburlino G., Taffetani F., Vagge I., Zitti S. & Zivkovic L., 2014. Plant communities of Italy: The Vegetation Prodrome. Plant Biosystem 148: 728-814. |
| *Cardamine chelidonia* L. | Gentile S., 1970. Sui faggeti dell’Italia meridionale. Atti Ist. Bot. Univ. Pavia serie 6, 5 (1969): 207-306.  Di Pietro R. 2009. Observations on the beech woodlands of the Apennines (peninsular Italy): an intricate biogeographical and syntaxonomical issue. Lazaroa 30: 89-97.  Biondi E., Blasi C., Allegrezza M., Anzellotti I., Azzella M.M., Carli E., Casavecchia S., Copiz R., Del Vico E., Facioni L., Galdenzi D., Gasparri R., Lasen C., Pesaresi S., Poldini L., Sburlino G., Taffetani F., Vagge I., Zitti S. & Zivkovic L., 2014. Plant communities of Italy: The Vegetation Prodrome. Plant Biosystem 148: 728-814. |
| *Doronicum columnae* Ten. | Dzwonko Z., Loster S., 2000. Syntaxonomy and phytogeographical differentiation of the Fagus woods in the Southwest Balkan Peninsula. J. Veg. Sci. 11: 667-678.  Fernandez Alvarez, 2003. Systematics of Eurasian and North African Doronicum (Asteraceae: Senecioneae). Annals of the Missouri Botanical Garden 90: 319-389. DOI: [*https://doi.org/10.2307/3298534*](https://doi.org/10.2307/3298534)  Biondi E., Blasi C., Allegrezza M., Anzellotti I., Azzella M.M., Carli E., Casavecchia S., Copiz R., Del Vico E., Facioni L., Galdenzi D., Gasparri R., Lasen C., Pesaresi S., Poldini L., Sburlino G., Taffetani F., Vagge I., Zitti S. & Zivkovic L., 2014. Plant communities of Italy: The Vegetation Prodrome. Plant Biosystem 148: 728-814.  Karadžić B., 2018. Beech forests (order Fagetalia sylvaticae Pawlowski 1928) in Serbia. Botanica Serbica 42 (1): 91-107. |
| *Doronicum orientale* Hoffm. | Gentile S., 1970. Sui faggeti dell’Italia meridionale. Atti Ist. Bot. Univ. Pavia serie 6, 5 (1969): 207-306.  Dzwonko Z., Loster S., 2000. Syntaxonomy and phytogeographical differentiation of the Fagus woods in the Southwest Balkan Peninsula. J. Veg. Sci. 11: 667-678.  Bergmeier E. & Dimopoulos P., 2001. Fagus sylvatica forest vegetation in Greece: Syntaxonomy and gradient analysis. J. Veg. Sci. 12: 109-126.  Fernandez Alvarez, 2003. Systematics of Eurasian and North African Doronicum (Asteraceae: Senecioneae). Annals of the Missouri Botanical Garden 90: 319-389. DOI: [*https://doi.org/10.2307/3298534*](https://doi.org/10.2307/3298534)  Di Pietro R. 2009. Observations on the beech woodlands of the Apennines (peninsular Italy): an intricate biogeographical and syntaxonomical issue. Lazaroa 30: 89-97.  Biondi E., Blasi C., Allegrezza M., Anzellotti I., Azzella M.M., Carli E., Casavecchia S., Copiz R., Del Vico E., Facioni L., Galdenzi D., Gasparri R., Lasen C., Pesaresi S., Poldini L., Sburlino G., Taffetani F., Vagge I., Zitti S. & Zivkovic L., 2014. Plant communities of Italy: The Vegetation Prodrome. Plant Biosystem 148: 728-814.  Karadžić B., 2018. Beech forests (order Fagetalia sylvaticae Pawlowski 1928) in Serbia. Botanica Serbica 42 (1): 91-107. |
| *Drymochloa drymeja* subsp. *exaltata* (C. Presl) Foggi & Signorini | Willner W, Di Pietro R, Bergmeier E. 2009. Phytogeographical evidence for post-glacial dispersal limitation of European beech forest species. Ecography 32:1011–1018.  Biondi E., Blasi C., Allegrezza M., Anzellotti I., Azzella M.M., Carli E., Casavecchia S., Copiz R., Del Vico E., Facioni L., Galdenzi D., Gasparri R., Lasen C., Pesaresi S., Poldini L., Sburlino G., Taffetani F., Vagge I., Zitti S. & Zivkovic L., 2014. Plant communities of Italy: The Vegetation Prodrome. Plant Biosystem 148: 728-814. |
| *Lamium flexuosum* Ten. | Willner W, Di Pietro R, Bergmeier E. 2009. Phytogeographical evidence for post-glacial dispersal limitation of European beech forest species. Ecography 32:1011–1018.  Biondi E., Blasi C., Allegrezza M., Anzellotti I., Azzella M.M., Carli E., Casavecchia S., Copiz R., Del Vico E., Facioni L., Galdenzi D., Gasparri R., Lasen C., Pesaresi S., Poldini L., Sburlino G., Taffetani F., Vagge I., Zitti S. & Zivkovic L., 2014. Plant communities of Italy: The Vegetation Prodrome. Plant Biosystem 148: 728-814. |
| *Myosotis sylvatica* Hoffm. subsp. *elongata* (Strobl) Grau | Biondi E., Blasi C., Allegrezza M., Anzellotti I., Azzella M.M., Carli E., Casavecchia S., Copiz R., Del Vico E., Facioni L., Galdenzi D., Gasparri R., Lasen C., Pesaresi S., Poldini L., Sburlino G., Taffetani F., Vagge I., Zitti S. & Zivkovic L., 2014. Plant communities of Italy: The Vegetation Prodrome. Plant Biosystem 148: 728-814. |
| *Oxalis acetosella* L. | Packhman J.R., 1978. Oxalis acetosella L. Biological flora of the British Isles. Journal of Ecology 66 (2): 669-693 |
| *Polystichum lonchitis* (L.) Roth | Dahl E., 1946. On different types of unglaciated areas during ice ages and their significance to phytogeography. New Phytol. 45: 225-242  Poldini L., Nardini S., 1993. Boschi di forra, faggete e abieteti in Friuli (NE Italia). Studia Geobotanica 3; 215-298.  Dzwonko Z., Loster S., 2000. Syntaxonomy and phytogeographical differentiation of the Fagus woods in the Southwest Balkan Peninsula. J. Veg. Sci. 11: 667-678.  Karadžić B., 2018. Beech forests (order Fagetalia sylvaticae Pawlowski 1928) in Serbia. Botanica Serbica 42 (1): 91-107. |
| *Ranunculus brutius* Ten. | Bergmeier E. & Dimopoulos P., 2001. Fagus sylvatica forest vegetation in Greece: Syntaxonomy and gradient analysis. J. Veg. Sci. 12: 109-126.  Willner W, Di Pietro R, Bergmeier E. 2009. Phytogeographical evidence for post-glacial dispersal limitation of European beech forest species. Ecography 32:1011–1018.  Biondi E., Blasi C., Allegrezza M., Anzellotti I., Azzella M.M., Carli E., Casavecchia S., Copiz R., Del Vico E., Facioni L., Galdenzi D., Gasparri R., Lasen C., Pesaresi S., Poldini L., Sburlino G., Taffetani F., Vagge I., Zitti S. & Zivkovic L., 2014. Plant communities of Italy: The Vegetation Prodrome. Plant Biosystem 148: 728-814. |

## Supplementary Figures


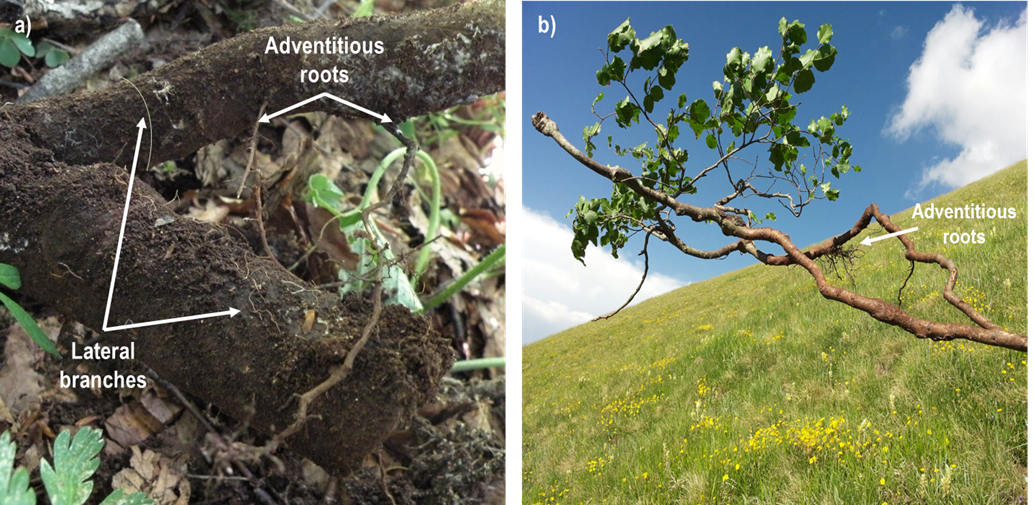


Supplementary Figure S1. Adventitious roots on procumbent basal lateral plagiotropic branches of *F. sylvatica* layered at a) 2,070 and b) 2,100 m a.s.l, respectively. Both pictures were taken on the 2020 summer season.


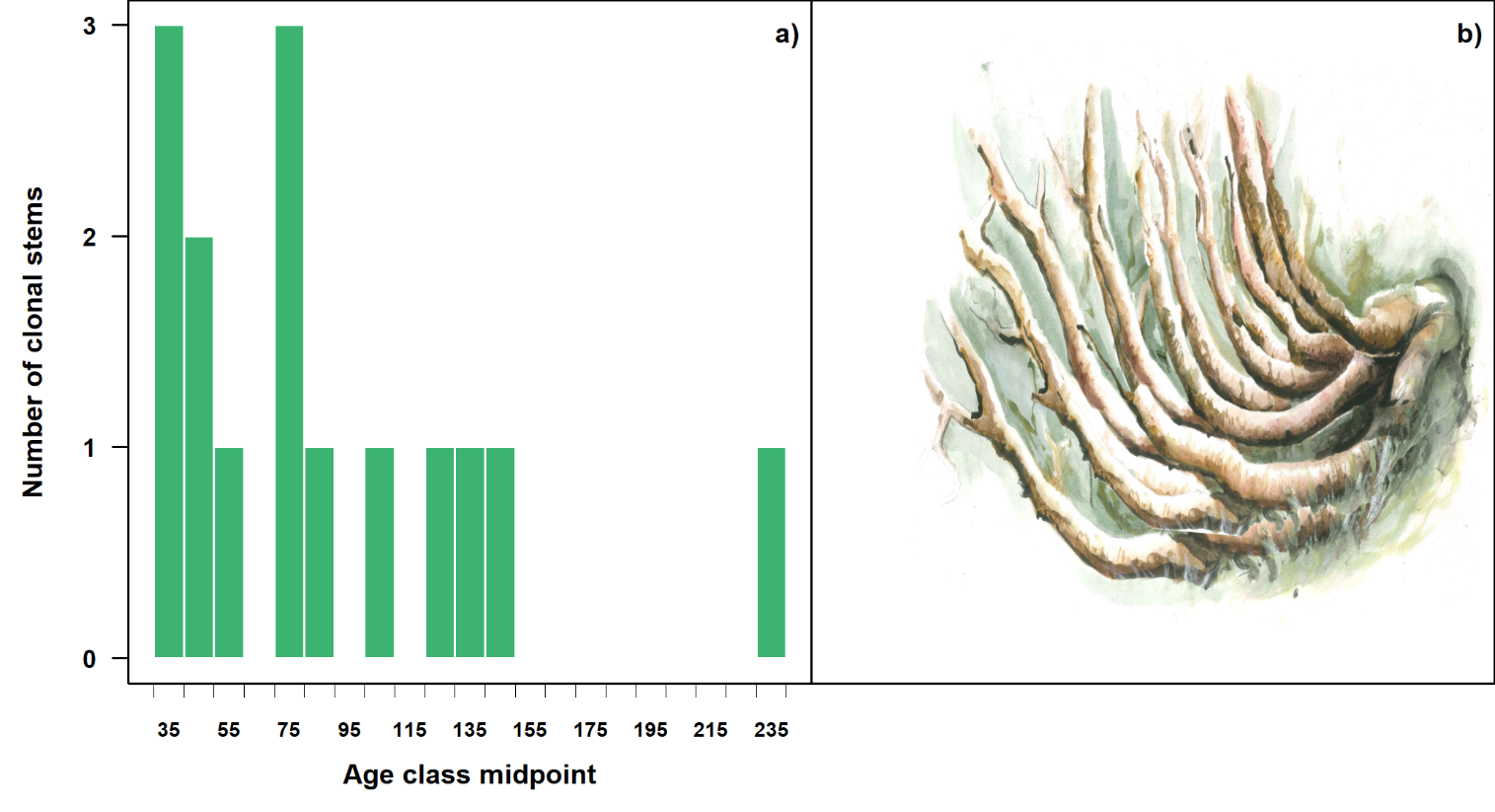


Supplementary Figure S2. Age class distribution of the *F. sylvatica* clonal stems growing at 2,040 m a.s.l. on the north-western slope of the Serra del Prete mountain. In a) absolute frequency distribution of clonal stems by age classes. Clonal stems are grouped in 10 years fixed-width age classes. In b) *F. sylvatica* polycormic profile with a number of 15 clonal stems presumably layered from a common ancestor genet.


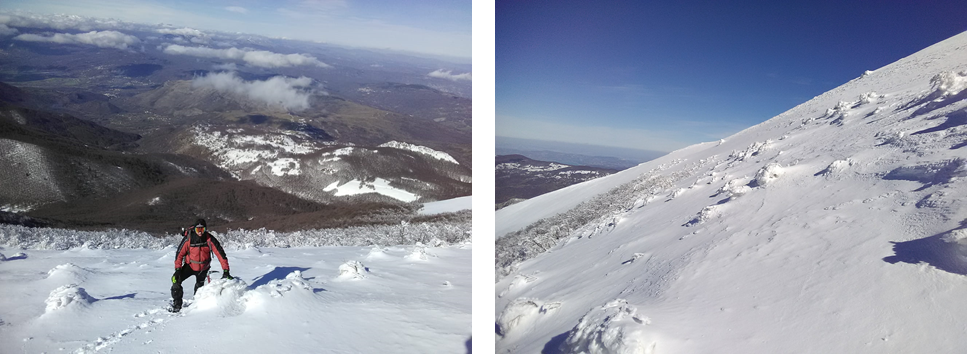


**Supplementary Figure S3.** *F. sylvatica* subalpine forest stand totally covered by snow at 2,130 m a.s.l. on the north-western flank of Serra del Prete peak (Pollino Massif). Both pictures were taken on the 2019 winter season.
